# Level and Contamination Assessment of Environmentally Sensitive Elements in Smaller than 100 μm Street Dust Particles from Xining, China

**DOI:** 10.3390/ijerph110302536

**Published:** 2014-02-28

**Authors:** Ni Zhao, Xinwei Lu, Shigang Chao

**Affiliations:** School of Tourism and Environment, Shaanxi Normal University, Xi’an 710062, China; E-Mails: zhaonikuaile@gmail.com (N.Z.); chaoshigang2012@163.com (S.C.)

**Keywords:** street dust, environmentally sensitive elements, enrichment factor, contamination assessment

## Abstract

Concentrations of the environmentally sensitive elements (ESEs) As, Co, Cu, Mn, Ni, Pb, V and Zn in smaller than 100 μm street dust particles from Xining were measured using X-ray fluorescence spectrometry and their contamination levels were assessed based on enrichment factor (*EF*), geoaccumulation index (*I*_geo_) and pollution load index (*PLI*). The concentrations of As, Co, Cu, Mn, Ni, Pb, V and Zn in smaller than 100 μm street dust particles from Xining are 0.1–0.8, 2.7–10.9, 0.7–5.2, 0.3–1.1, 0.6–2.5, 1.2–11.1, 0.7–1.3 and 0.4–2.9 times the background values of Qinghai soil, respectively. The calculated *EF* and *I*_geo_ values reveal the order Co > Pb > Cu > Zn > V > Ni > Mn > As. The *EF* and *I*_geo_ values of Co, Cu, Pb and Zn are higher indicating that there is considerable pollution by these elements in smaller than 100 μm street dust particles, especially for Co. The *EF* and *I*_geo_ of Mn, Ni and V are lower and the assessment results indicate an absence of distinct Mn, Ni and V pollution in the studied samples. The mean value of *PLI*_site_ is 1.14, indicating a slightly pollution in the whole city of Xining. The order of *PLI*_area_ for the five tested districts is Center District (CD) > East District (ED) > West District (WD) > North District (ND) > South District (SD), showing that ESEs pollution in the South District is the lightest while it is the highest in the Central District.

## 1. Introduction

At present, nearly half of the world’s population lives in urban agglomerations [1], and the dense population leads to an increasing amount of pollutants being discharged into the urban environment. Streets, an essential component of the urban landscape, are conspicuous sources of dissolved and sediment-associated contaminants [2,3,4]. Street dust, particles deposited on roads, is an important medium hosting environmental pollutants in urban environments [5,6], and can easily accumulate in the human body via directly inhalation, ingestion and dermal contact absorption [7,8,9,10]. Dust particles can migrate via saltation (diameters > 500 μm), creep (100 μm < diameters < 500 μm), suspension and re-suspension (diameters < 100 μm) [11]. The re-suspension of street dust has proven to be an important contributor to atmospheric particulate matter in urban areas [12,13], and these polluted particles are easily deposited with a greater dry and wet deposition scope after being spread broadly [14].

Contaminants in street dust include inorganic toxic metals, de-icing salts, and organic compounds such as polycyclic aromatic hydrocarbons (PAHs), polychlorinated biphenyls (PCBs), and pesticides [15]. The concentration of toxic metals in street dust has proven to be extremely variable [16]. These toxic metals such as As, Co, Cu, Mn, Ni, Pb, V and Zn are also collectively called environmentally sensitive elements (ESEs) [17]. In urban areas, street dust, which serves as both a sink and source for ESEs [18,19,20], is emitted from mobile or stationary sources [7,21,22,23,24,25], such as vehicle wear, industrial activities, domestic heating, activities of construction and demolition, degradation of road paint and waste incineration. The ESEs in street dust may harm the urban environment and endanger the ecosystem’s health.

In recent decades many studies on street dust have focused on the concentration, distribution and source identification of ESEs [8,22,26,27,28,29]. Environmental quality and potential health risk assessment methods have been applied to explore the potential harm caused by ESEs from street dust [30,31]. Contamination levels of ESEs in street dust from different cities or different functional areas of city have also been compared [5,28,32]. The dispersion and distribution of ESEs are highly dependent on the size of particles and the surface properties of the substrate on which ESEs are deposited [33]. ESEs are preferentially presented in finer particles [18,34], due to their lower density and greater surface area [34]. ESEs in the finer fraction could have greater effects on human health due to the fact they remain for a longer time in air, adhere to the skin and are inhaled through the nose or mouth more easily [30]. Fine street dust particulates are not always removed efficiently through street sweeping, which as a basic contaminant control measure [35]. For example, only 50% of all fractions of road deposited sediments could be removed by sweeping and the removal rate for dust particles of diameter <104 μm is only 15%–20% [36,37].

Former studies focused on ESEs in street dust with diameters ranging from 0.1 to 2,000 μm with most being higher than 100 μm [24,25,38,39,40,41,42,43]. However, the research about contamination level of ESEs in smaller than 100 μm street dust particles is lacking, especially in the northwest cities of China. The particles smaller than 100 μm in street dust have a greater impact on the environment and human health. The objectives of the present work were thus to determine the concentration levels of the ESEs As, Co, Cu, Mn, Ni, Pb, V and Zn in smaller than 100 μm street dust particles from Xining, China and to assess their contamination level. The results could be useful for regulators and engineers involved in environmental protection and management. 

## 2. Materials and Methods

### 2.1. Study Area

**Figure 1:**
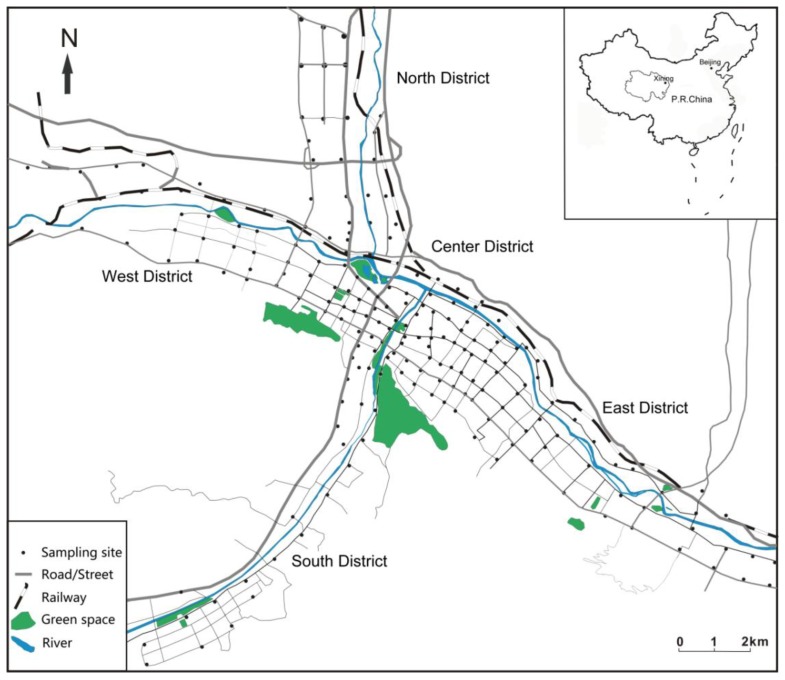
Study area and sampling sites in Xining.

Xining, the capital of Qinghai Province, is located in the eastern Tibetan Plateau with the longitude 101°77'E and latitude 36°62'N (Figure 1). Xining City has a typical continental plateau semi-arid climate with an annual temperature of −18.9 to 34.6 °C, annual average rainfall of 380 mm and annual average evaporation of 1,360 mm. Xining is surrounded by mountains with the average altitude is 2,260 m and three rivers converge on the city. The prevailing wind direction in Xining is northwest and the northwest altitude is high, while the southeast one is low. The urban population of Xining was 941,000 in 2000 and it had increased to 1,198,000 by 2010. Meanwhile the urbanization rate was 59.59% in 2006 and it increased to 67.73% in 2012. There were more than 300,000 motor vehicles in Xining in February 2013, while the number of motor vehicles was only 100,000 in 2006. The urban construction area of Xining is 75 km^2^. Xining urban area consists of five areas, *i*.*e*., East District (ED), West District (WD), South District (SD), North District (ND) and Center District (CD). 

Dongchuan industrial park is located in ED which is a new materials, new energy and mechanical processing area. Xining steel group, the railway station and bus terminal are located in WD. South Hill and Nanshan park are located in SD. There is one large steel market located in ND. CD is a commercial and residential mixed area with crowded traffic and dense population. All streets in Xining city are cleaned by sweeping every day in the morning. 

### 2.2. Sampling and Analytical Methods

Dust sampling sites covered all five districts of the Xining urban area (Figure 1). At every sampling site, dust samples were collected by sweeping with a clean plastic brush and a dustpan [25,39] from five to eight points of the road or pavement edges twice during the dry season in July 2012. The amount street dust collected at each sampling point was about 30–50 g/m^2^. The twice collected samples were mixed to form a composite sample of ~500 g representing each sampling site and stored in a sealed polyethylene bag. In the laboratory, all the samples were air-dried naturally and sieved through a 100 μm nylon sieve. The fraction below 100 μm particle was collected. The concentrations of As, Co, Cu, Mn, Ni, Pb, V and Zn in the smaller than 100 μm street dust particles were measured by using wavelength dispersive X-ray fluorescence spectrometry (XRF, PW2403 apparatus, PANalytical, Almelo, Netherlands) [39]. Standard samples (GSD-12, GSS10) and 15% of the repeat samples were used for quality control in the experiments. The analytical precision, measured as relative standard deviation, was routinely 3%–5%. Accuracy of the analyses was checked using standard and duplicate samples. The quality control gave good precision (S.D. < 5%).

### 2.3. Methods of Contamination Assessment

A number of calculation methods have been put forward for quantifying the degree of ESEs’ enrichment or pollution in dust [6,16,44]. In this study, geoaccumulation index (*I_geo_*), enrichment factor (*EF*) and pollution load index (*PLI*) are calculated to assess the metal contamination levels in the studied samples.

#### 2.3.1. Enrichment Factor

For each ESE (*i*), the enrichment factor (*EF*) is defined in Equation (1):

*EF* = (*C_i_* / *C_ref_* )*_sample_* /(*C_i_* / *C_ref_*)*_background_* (1)

where (*C_i_*/*C_ref_*) is the ratio of concentration of metal *i* to the concentration of a reference metal in the sample and background. *EF* of an element in the studied samples is based on the standardization of a measured element against a reference element. A reference element is often the one characterized by low occurrence of variability, such as the most commonly used elements: Al, Fe, Ti, Si, Zr, *etc*. [38,45,46,47]. Five different categories defined for *EF* values [38,45] are listed in Table 1.

**Table 1:** Pollution index and pollution grade.

EF	Enrichment Category	I_geo_	Pollution Category	PLI	Pollution Category
EF < 2	Deficiency to minimal polluted	I_geo_ ≤ 0	Unpolluted	0 < PLI ≤ 1	Unpolluted
2 ≤ EF < 5	Moderate polluted	0 < I_geo_ ≤ 1	Unpolluted to moderately polluted	1 < PLI ≤ 2	Unpolluted to moderately
5 ≤ EF < 20	Significant polluted	1 < I_geo_ ≤ 2	Moderately polluted	2 < PLI ≤ 3	Moderately polluted
20 ≤ EF < 40	Very high polluted	2 < I_geo_ ≤ 3	Moderately to strongly polluted	3 < PLI ≤ 4	Moderately to highly polluted
EF > 40	Extremely high polluted	3 < I_geo_ ≤ 4	Strongly polluted	4 < PLI ≤ 5	Highly polluted
		4 < I_geo_ ≤ 5	Strongly to extremely polluted	PLI > 5	Very highly polluted
		I_geo_ > 5	Extremely polluted		

#### 2.3.2. Geoaccumulation Index

The geoaccumulation index (*I_geo_*) was originally defined by Müller [24] and used for bottom sediments. By making comparisons with pre-industrial levels, *I_geo_* is capable of evaluating pollutant accumulation. It is computed through the Equation (2):

*I_geo_* = log_2_ (*C_i_* / (1.5 *B_i_*)) (2)

where *C_i_* represents the measured concentration of the element *i* and *B_i_* is the geochemical background value of the element *i* in fossil argillaceous sediment (average shale). In the study, *B_i_* is the background value of element *i* in Qinghai soil [48]. The factor 1.5 is introduced in this equation to minimize the effect of possible variations in the background values. The geoaccumulation index for each ESE is classified as shown in Table 1.

#### 2.3.3. Pollution Load Index

In order to assess the integrative impact of anthropogenic activity on related ESEs, the Tomlinson multi-element pollution load index (*PLI*) [49] is calculated based on each element concentration (As, Co, Cu, Ni, Mn, Pb, V and Zn). The *PLI* index for each sampling site is defined as Equation (3):

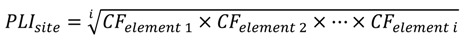
(3)
where the *CF_element_* *_i_* is the ratio between the content of metal *i* to its background value, which is computed with Equation (4):

*CF_elementi_= C_i_ / B_i_* (4)

where *C_i_* represents the measured concentration of the element *i* and *B_i_* is the background value of element *i* in Qinghai soil [48]. Finally, individual *PLI*_site_ values were used to compute *PLI*_area_ value for each sampling area as Equation (5) [15]:

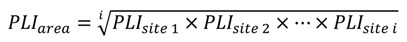
(5)
where *i* is the number of the sampling site in each sampling area including East District (ED), West District (WD), South District (SD), North District (ND), Central District (CD) and Total Districts (TD). The *PLI* index is classified as shown in Table 1.

## 3. Results and Discussion

### 3.1. Concentration of ESEs in Smaller than 100 μm Street Dust Particles

**Figure 2:**
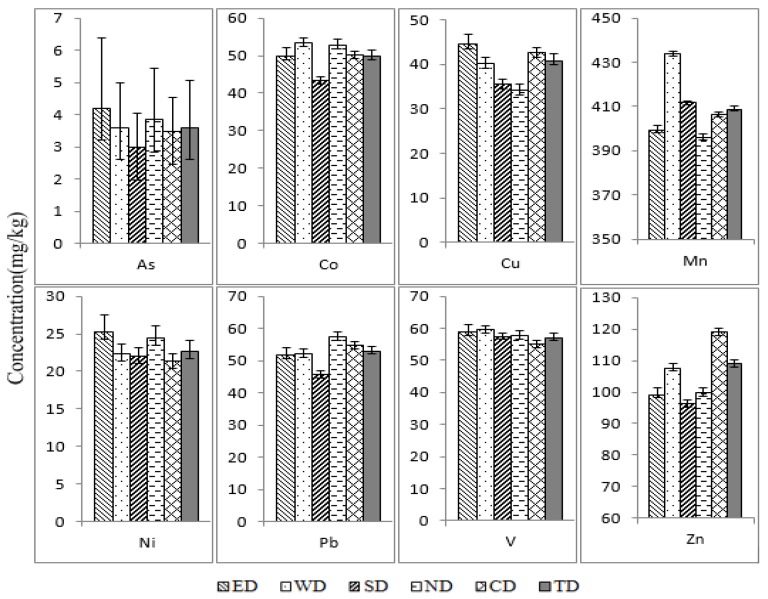
Concentrations of ESEs in smaller than 100 μm street dust particles from Xining (ED: East District; WD: West District; SD: South District; ND: North District; CD: Central District; TD: Total District).

Descriptive statistical values of ESEs concentrations in smaller than 100 μm street dust particles from Xining are summarized in Table 2. The background values for Qinghai soil [48], listed in Table 2, are used as reference values. The concentrations of ESEs in smaller than 100 μm street dust particles from the different districts, *i*.*e*., East District (ED), West District (WD), South District (SD), North District (ND) and Central District (CD), as well as the Total District (TD) are shown in Figure 2.

**Table 2:** Concentrations of ESEs in smaller than 100 μm street dust particles from Xining (mg/kg).

Elements	Min	5%	25%	50%	Mean	75%	95%	Max	S.D.	Kurtosis	Skewness	Reference Value
As	0.8	1.8	2.7	3.3	3.6	4.1	6.7	11.1	1.5	5.9	1.8	14.0
Co	27.2	34.3	41.5	49.7	50.0	56.5	67.3	110.2	11.3	3.6	1.0	10.1
Cu	15.1	20.2	30.5	37.9	40.8	47.5	76.6	115.2	16.4	3.5	1.5	22.2
Mn	150.1	351.9	385.8	405.7	408.7	425.1	506.2	623.5	47.7	8.2	0.3	580.0
Ni	16.8	18.3	19.8	21.2	22.6	22.6	30.9	74.1	6.6	30.6	5.0	29.6
Pb	24.4	34.8	41.5	48.5	52.9	56.6	85.8	233.0	21.0	29.5	4.3	20.9
V	47.4	51.5	53.6	55.5	57.1	58.7	65.2	96.8	6.0	14.6	3.0	71.8
Zn	33.1	64.4	85.9	106.9	108.9	127.1	161.8	231.1	31.3	0.9	0.6	80.3

As shown in Table 2, the mean concentration of As, Co, Cu, Mn, Ni, Pb, V and Zn in smaller than 100 μm street dust particles from Xining City is 3.6, 50.0, 40.8, 408.7, 22.6, 52.9, 57.1 and 108.9 mg/kg, respectively. Based on the mean metal concentrations in the studied samples, the ratios of mean concentration and the reference value is in order Co > Pb > Cu > Zn > V > Ni > Mn > As. The mean concentrations of Co, Cu, Pb and Zn in smaller than 100 μm street dust particles are significantly higher than their background values of Qinghai soil, indicating possible contamination, likely caused by an anthropogenic source. The concentrations of As, Co, Cu, Mn, Ni, Pb, V and Zn in the smaller than 100 μm street dust particles are 0.1–0.8, 2.7–10.9, 0.7–5.2, 0.36–1.1, 0.6–2.5, 1.2–11.1, 0.7–1.3 and 0.4–2.9 times the background value of Qinghai soil, respectively. The 95th percentile values of As, Mn, Ni and V are lower or slightly higher than their background values and their mean concentrations are 0.5, 0.9, 1.0 and 0.9 times the background values, respectively, indicating that these four metals may mainly originate from natural sources. The 25th percentile values of Co, Cu, Pb and Zn are obviously higher than their corresponding background values, indicating they may be influenced by the anthropogenic pollution. Similar results have been reported before, showing that Pb, Zn and Cu in the street dust of Baoji were 5–71, 5–24 and 3–12 times the background values of Shaanxi soil [25]. The mean concentrations of Co, Cu, Pb and Zn in re-suspended dust from Xining are 5.0, 1.8, 2.5 and 1.4 times the background values, respectively, so we should pay more attention to their potential risks to local humans [44]. 

Figure 2 shows the concentrations of ESEs in smaller than 100 μm street dust particles from the different districts. The mean concentration orders for each metal in the different districts is As: ED > ND > WD > CD > SD; Co: WD > ND > CD > ED > SD; Cu: ED > CD > WD > SD > ND; Mn: WD > SD > CD > ED > ND; Ni: ED > ND > WD > SD > CD; Pb: ND > CD > WD > ED > SD; V: WD > ED > SD > ND > CD; Zn: CD > WD > ED > ND >SD. The highest concentrations of Co, Cu, Pb and Zn in the studied samples were found in WD, ED, ND and CD, respectively, indicating these four metals may derive from different anthropogenic sources due to the different regional characteristics of the four districts.

### 3.2. Assessment Results of the ESEs Contamination in Smaller than 100 μm Street Dust Particles

#### 3.2.1. Enrichment Index Assessment Results

**Figure 3:**
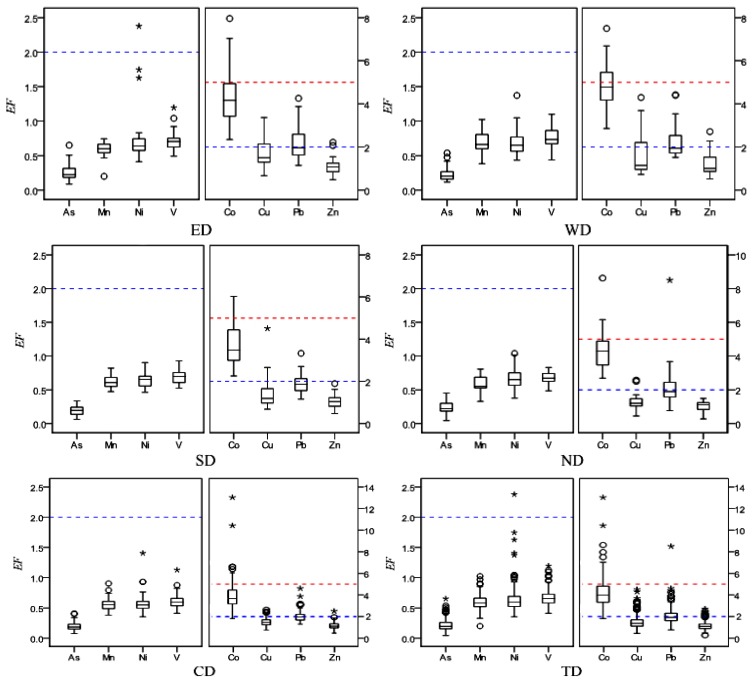
Box-plots of *EF* for ESEs in smaller than 100 μm street dust particles from Xining (ED: East District; WD: West District; SD: South District; ND: North District; CD: Central District; TD: Total District; **°**: outlier; *: extreme).

Enrichment factors of ESEs are calculated for each metal relative to the background value of Qinghai soil [48]. In this study, Zr is used as a reference element. The Box-plots of *EF* for ESEs in smaller than 100 μm street dust particles from Xining are provided in Figure 3.

The *EF* values of As, Co, Cu, Mn, Ni, Pb, V and Zn in the smaller than 100 μm street dust particles are in the range of 0.04–0.65, 1.82–13.03, 0.45–4.51, 0.2–1.02, 0.35–2.38, 0.77–8.5, 0.41–1.19 and 0.27–2.71, with an average of 0.22, 4.19, 1.53, 0.59, 0.64, 2.10, 0.67 and 1.14, respectively. All the *EF* values of As, Mn, Ni and V are lower than 2, indicating these four metals are in the deficiency to minimal pollution range, according to the pollution grade ratings in Table 1, while for Cu, Pb and Zn, 84%, 55% and 97% the *EF* values are lower than 2, and 16%, 45% and 3% *EF* values are between 2 and 5, respectively, indicating they are minimal to moderate pollutants. Co has 78% *EF* in the 2 to 5 and 21% *EF* in the 5 to 20 range, indicating the Co is moderate to significant polluted. The order of mean *EF* values is Co (4.19) > Pb (2.10) > Cu (1.53) > Zn (1.14) > V (0.67) > Ni (0.64) > Mn (0.59) > As (0.22).

The *EF* values of the ESEs in each district are different. The highest mean *EF* values of Co (4.89) and Zn (1.23) are found in the West District (WD), where the Xining steel group, railway station and bus terminal are located. Co and Zn can be found in stainless and alloy steels [50] and Co in dust appears to come at least partially from automotive emissions [25], Zn has a traffic source coupled with industrial sources [39]. The highest mean *EF* values of Cu and Pb occurred in the East District (ED) and North District (ND), respectively, where the main traffic and industrial areas are, indicating these two elements are strongly influenced by the mechanical abrasion of vehicle parts [51] and industrial pollution [39]. The *EF* values of all analyzed metals in smaller than 100 μm street dust particles from the South District (SD) are the lowest, indicating the South District of Xining is barely influenced by human activities.

#### 3.2.2. Assessment Results of Geoaccumulation Index

**Figure 4:**
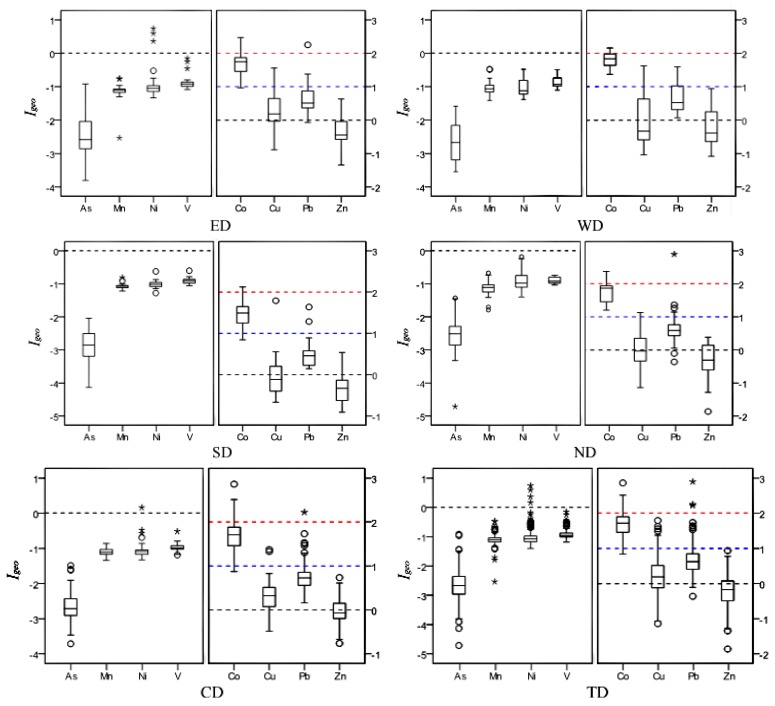
Box-plots of *I_geo_* for ESEs in smaller than 100 μm street dust particles from Xining (ED: East District; WD: West District; SD: South District; ND: North District; CD: Central District; TD: Total District; **°**: outlier; *: extreme).

The calculated results of *I*_geo_ of ESEs in smaller than 100 μm street dust particles from Xining are presented in Figure 4. The *I*_geo_ values of As, Co, Cu, Mn, Ni, Pb, V and Zn in the investigated samples range from −0.47 to −0.92, 0.84 to 2.86, −1.14 to 1.79, −2.54 to −0.48, −1.40 to 0.74, −0.36 to 2.89, −1.18 to −0.15 and −1.86 to 0.94, with an average of −2.64, 1.69, 0.19, −1.10, −1.01, 0.68, −0.92 and −0.21, respectively. The order of mean *I*_geo_ is Co (1.69) > Pb (0.68) > Cu (0.19) > Zn (−0.21) > V (−0.92) > Ni (−1.01) > Mn (−1.10) > As (−2.64), similar to the order of *EF*. All *I*_geo_ values for As, Mn and Ni and 98% *I*_geo_ of V are less than 0, indicating that the studied samples in Xining is unpolluted by As, Mn, V and Ni. 

The mean *I_geo_* value and 69% *I_geo_* values of Zn are less than 0 (unpolluted), and 31% *I_geo_* values of Zn are slightly higher than 0, indicating an unpolluted to moderately polluted status. The 58% *I_geo_* values of Cu and 80% *I_geo_* values of Pb, as well as the mean *I_geo_* values of Cu and Pb, are between 0 and 1 revealing an unpolluted to moderately polluted status, while 34% *I_geo_* values of Pb and 2% *I_geo_* values of Cu are lower than 0 and 8% *I_geo_* values of Pb and 16% *I_geo_* values of Cu are between 1 and 2, revealing the unpolluted or moderately polluted status of these metals. The 84% and 14% *I_geo_* values of Co fall into 1 to 2 and 2 to 3, respectively, and only 2% are between 0 and 1, indicating Co is a moderate pollutant and moderate to strong pollutant. The *I_geo_* results indicates that the smaller than 100 μm street dust particles from Xining City are contaminated by with different levels of different ESEs, especially Co, Cu, Pb and Zn, which are derived from anthropogenic sources. 

The *I_geo_* results in each district are same as the *EF* results except for Zn. The highest mean *I_geo_* value of Zn is found in the Central District, while it is in the West District in the *EF* analysis, which may be related to the concentration of reference element Zr in these two districts. The Central District is a commercial and residential mixed zone which is influenced by heavy traffic and human activities, which further explains a significant contribution to Zn and Pb from traffic-related sources [7,51].

#### 3.2.3. Pollution Load Index Assessment Results

**Figure 5:**
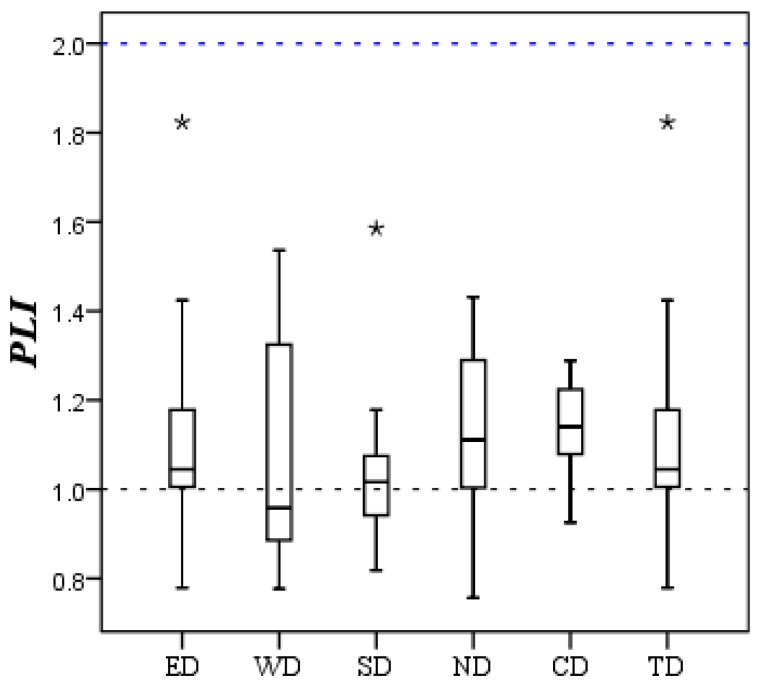
Box-plots of *PLI* for ESEs in smaller than 100 μm street dust particles from Xining (ED: East District; WD: West District; SD: South District; ND: North District; CD: Central District; TD: Total District; *: extreme).

The calculated *PLI* values including eight ESEs (As, Co, Cu, Mn, Ni, Pb, V and Zn) in smaller than 100 μm street dust particles from Xining are summarized in Figure 5. According to Suresh *et al*. [52], *PLI* values equal to zero indicate perfection, a value of one indicates baseline levels of pollutants present and values above one indicate progressive deterioration. The extent of pollution increases with the increase in the numerical *PLI* value.

Figure 5 shows that *PLI_site_* values range from 0.76 to 1.82, with a mean value of 1.14 in the whole city of Xining (TD). 20% *PLI_site_* values in Xining are lower than one, indicating an unpolluted status, but 80% *PLI_site_* values are between 1 and 2, indicating most street dust sampling sites are unpolluted to moderately polluted. Three higher *PLI_site_* values (1.82, 1.75 and 1.65) are found in the samples collected from heavy traffic and industrial areas. The overall *PLI_area_* value for Xining is calculated and the result is 1.12, indicating a slightly moderately polluted status.

As for the five districts in Xining City, the *PLI_area_* is 1.14, 1.13, 1.03, 1.11 and 1.15 for ED, WD, SD, ND and CD, respectively. The values of the five areas are roughly the same, and slightly higher than one. South District (SD) has the lowest *PLI_area_* value among five districts, and is where South Hill and Nanshan Park are located; these indicate that South District is seldom affected by the human activities such as heavy traffic and industries. The highest *PLI_area_* value occurred in the Central District (CD), which is the commercial and residential mixed zone, easily influenced by anthropogenic sources.

## 4. Conclusions

Street dust samples were collected from Xining and the concentrations of ESEs As, Co, Cu, Mn, Ni, Pb, V and Zn in the smaller than 100 μm street dust particle samples were determined using the XRF method. The results indicate that Co, Cu, Pb and Zn were significantly concentrated in the smaller than 100 μm street dust particles from Xining City. Their *EF* and *I_geo_* values reveal that Co, Cu, Pb and Zn in the studied samples presented different level of contamination, while As, Mn, Ni and V were non-pollutants. The pollution load index assessment results indicate that the smaller than 100 μm street dust particles from Xining presented a slightly polluted status as a whole, and ESE pollution in the South District is lighter than in other districts. The environmental risk of ESEs As, Co, Cu, Mn, Ni, Pb, V and Zn in the smaller than 100 μm street dust particles is not only related to their respective concentration levels, but with their speciation in the samples. The speciation of ESEs in the smaller than 100 μm street dust particles and their health risk will be further investigated in the future work. 
